# Pilot study of losartan for pulmonary hypertension in chronic obstructive pulmonary disease

**DOI:** 10.1186/1465-9921-6-88

**Published:** 2005-08-01

**Authors:** Nicholas W Morrell, Matthew A Higham, Peter G Phillips, B Haleema Shakur, Paul J Robinson, Ray J Beddoes

**Affiliations:** 1 Department of Respiratory Medicine, Imperial College School of Medicine, London, UK; 2 Medical Department, Merck Sharp & Dohme Limited, Hoddesdon, UK

## Abstract

**Background:**

Morbidity in COPD results from a combination of factors including hypoxia-induced pulmonary hypertension, in part due to pulmonary vascular remodelling. Animal studies suggest a role of angiotensin II and acute studies in man concur. Whether chronic angiotensin-II blockade is beneficial is unknown. We studied the effects of an angiotensin-II antagonist losartan, on haemodynamic variables, exercise capacity and symptoms.

**Methods:**

This was a double-blind, randomized, parallel group, placebo- controlled study of 48 weeks duration. Forty patients with COPD and pulmonary hypertension (Tran tricuspid pressure gradient (TTPG) = 30 mmHg) were randomised to losartan 50 mg or placebo. Changes in TTPG were assessed at 3, 6 and 12 months.

**Results:**

There was a trend for TTPG to increase in the placebo group (baseline 43.4 versus 48.4 mmHg at endpoint) and stay constant in the losartan group (baseline 42.8 versus 43.6 mmHg). More patients in the losartan group (50%) than in the placebo group (22%) showed a clinically meaningful reduction in TTPG at any timepoint; these effects seemed more marked in patients with higher baseline TTPG. There were no clear improvements in exercise capacity or symptoms.

**Conclusion:**

In this 12-month pilot study, losartan 50 mg had no statistically significant beneficial effect on TTPG, exercise capacity or symptoms in pulmonary hypertension secondary to obstructive disease. A sub-group of patients with higher TTPG may benefit.

## Background

Pulmonary hypertension is the main cardiovascular complication of chronic obstructive pulmonary disease (COPD) and is associated with a substantial increase in morbidity and mortality [1-3]. The major characteristic of COPD is chronic airflow limitation that progresses slowly over a period of years and is, by definition, largely irreversible [4]. We previously reported echocardiographic evidence of pulmonary hypertension in over 50% of patients attending a hospital clinic for COPD [5], with the highest prevalence in those with severe disease.

Alveolar hypoxia undoubtedly contributes to pulmonary hypertension in COPD and there is a correlation, albeit poor, between the pulmonary artery pressure and the degree of arterial hypoxaemia [6,7]. Structural remodelling of the pulmonary vasculature as a consequence of chronic alveolar hypoxia is probably the main contributor to the pathogenesis of pulmonary hypertension in COPD [3,8] although a number of other factors may be involved, including hyperinflation and loss of alveolar capillaries in emphysema [9]. In addition, a degree of structural remodelling of the distal pulmonary circulation has been demonstrated in smokers with mild airflow obstruction [10]. Although overt right heart failure is unusual in stable severe COPD, electrocardiographic signs of right heart dysfunction predict a higher mortality [11]. Moreover, pulmonary arterial pressure rises markedly in COPD patients on minimal exertion and it is likely that pulmonary hypertension contributes partly to exercise limitation in these patients. At present, there is no specific pharmacological therapy for pulmonary hypertension in COPD. Long-term oxygen therapy improves survival but does not reverse the pulmonary vascular changes [12].

A possible therapeutic role for losartan, a selective angiotensin-II antagonist, in hypoxic pulmonary hypertension has been suggested by results from animal models [13] and acute studies in man [14-17]. Losartan, which selectively binds to the AT_1 _receptor, is currently used for reducing central pressures in patients with heart failure [18] and is effective in reducing systemic hypertension. It has also been shown to regress carotid artery hypertrophy [19] and left ventricular hypertrophy [20] and to reduce cardiovascular mortality/morbidity [20] in hypertension.

We hypothesized that long-term administration of losartan may benefit patients with pulmonary hypertension secondary to COPD, using echo-Doppler derived measurements of Tran tricuspid pressure gradient (TTPG) as an index of pulmonary hypertension. We, therefore, undertook a study to evaluate the effects of losartan on TTPG, exercise capacity, quality of life, arterial blood gases and safety in patients with cor pulmonale secondary to severe COPD.

## Methods

### Patient Selection

Male or female patients, aged 50–80 years, were included. Patients had a clinical history of COPD, evidence of obstructive spirometry (FEV_1_/FVC ratio ≤ 70%), echocardiographic evidence of pulmonary arterial hypertension (TTPG ≥ 30 mmHg) and sitting systolic blood pressure ≥ 100 mmHg. Exclusion criteria included left ventricular dysfunction (ejection fraction <35%), myocardial infarction, significant renal impairment, recent infective exacerbation of COPD, or concomitant use of vasodilators, β-blockers or potassium-sparing diuretics. Patients were permitted to continue on their regular COPD therapy.

### Study design

This pilot study was conducted as a single centre, double-blind, randomized, parallel group comparison of losartan and placebo in patients with pulmonary hypertension secondary to COPD. Following the initial 4-week run-in phase, eligible patients were randomized (week 0) to receive either losartan (Cozaar, Merck & Co, NJ) or placebo for 48 weeks. Losartan 25 mg or a placebo tablet was administered once daily for 1 week. The dose was then increased to 50 mg daily (or placebo equivalent), providing the patient's systolic blood pressure remained ≥ 100 mmHg. The dose could be down titrated once (to 25 mg) if necessary.

The study protocol was approved by the hospital's research ethics committee, and all patients provided written informed consent to study participation.

### Study procedures

At the initial visit (week -4) patients underwent physical examination, spirometry, a practice exercise test (see below), echocardiography and safety blood/urine tests. For all eligible patients, tests were repeated at the baseline visit (week 0) when arterial blood gases were also measured.

Patients returned to the clinic at weeks 1, 4, 12, 24, 36 and 48 when blood pressure measurements and safety tests were repeated. Spirometry, echocardiography and exercise testing using a symptom-limited 10 m shuttle walk test [21] were repeated at weeks 12, 24 and at the end of the study (week 48) when arterial gases were sampled again. Echocardiographic assessments were carried out using a Toshiba Powervision model SSA380 ultrasound scanner and a multifrequency probe with a range of 2.5–3.7 MHz (Toshiba Medical Systems, West Sussex, UK). Mean maximum tricuspid valve regurgitation velocity (V) was recorded in m.s^-1 ^and used to calculate the TTPG in mmHg, as previously described [5]. Pulmonary hypertension was defined as a TTPG = 30 mmHg. Right atrial pressure (RAP) was estimated clinically from the height of the jugular venous pressure above the sternal angle, plus 5 cm (mean distance from RA to sternal angle), divided by 1.3 to convert to mmHg. Right ventricular systolic pressure could then be derived (TTPG + RAP).

A quality of life questionnaire (St George's Hospital Respiratory Questionnaire) [22] and Patient Health Survey (SF-36) were performed at weeks 0, 12, 24 and 48. Adverse experiences were monitored throughout the study.

### Outcome measures

The primary endpoint was change from baseline (week 0) in TTPG. Pre-specified secondary endpoints included change from baseline in other echocardiographic parameters including right ventricular systolic pressure, peak tricuspid regurgitant velocity, left ventricular fractional shortening and right ventricular wall thickness.

Change from baseline in exercise capacity, breathlessness score after exercise (on a 10 point visual analogue scale: 0 = best, 10 = worst); and quality of life assessments were specified as secondary outcomes.

### Statistical methods

This pilot study was designed with a sample size of 44 patients, to give a power of 85% to detect a 25% reduction in TTPG, assuming a baseline TTPG of 46 mmHg increasing by 3 mmHg in the placebo group during the year of the study and a standard deviation of 16 mmHg. This power calculation was based on studies of pulmonary arterial pressure estimated from measurement of TTPG in patients of a similar type [23], and the mean annual rate of increase in pulmonary hypertension in patients affected by COPD [3,24] A reduction of 25% from baseline was chosen as clinically relevant, based on previous studies on vasodilators [25,26] in pulmonary hypertension and therefore a magnitude worthy of further study. Analysis was based on intention-to-treat with last post-randomisation observation carried forward to study end where data were missing. Continuous efficacy variables were analysed by ANOVA. The validity of the assumptions for the ANOVA was confirmed from a review of plots of the residuals against predicted values.

## Results

**Figure 1:**
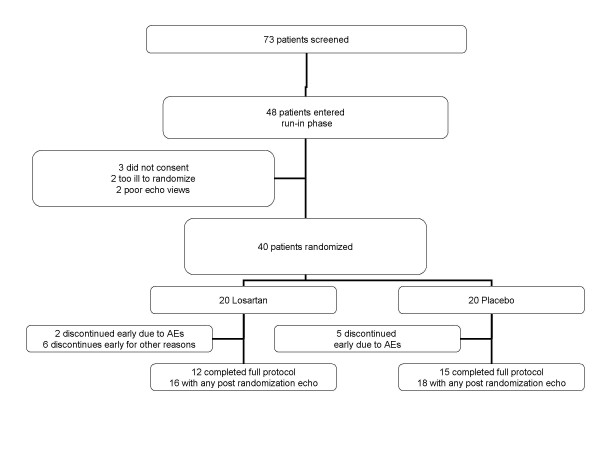
Overview of study design, randomization and drop-out rates.

A total of 73 patients with COPD underwent screening echocardiography. Of these, 48 patients entered the run-in phase of the study and of these, 20 were randomized to losartan and 20 to placebo (Figure 1). The 21 females and 19 males ranged in age from 52–79 years (mean 67 years). All had a clinical history of COPD of at least 1-year duration (mean 8.4 years); the mean baseline FEV_1 _was 0.83 L (range 0.28–1.95 L), with the mean percent of predicted FEV_1 _being 35.3%. The mean FEV_1_/FVC ratio was 36% (range 14 to 69%) The distribution of patient characteristics, symptoms and clinical data at baseline (Table 1) revealed no clinically meaningful differences between treatment groups.

**Table 1:** Patient characteristics at baseline – mean (SD)

	**Losartan (n = 20)**	**Placebo (n = 20)**
	Demography	
Male:Female	9:11	10:10
Age (years)	68 (8.4)	66 (7.3)
	Spirometry	
FEV_1 _(litres)	0.85 (0.40)	0.82 (0.38)
FVC (litres)	2.5 (0.9)	2.5 (0.9)
Percent predicted FEV_1 _(%)	37 (19)	33 (14)
	Echo findings	
TTPG (mmHg)	42.8 (8.8)	43.4 (9.9)
Estimated right atrial pressure (mmHg)	4.0 (0.8)	4.11 (0.9)
	Exercise capacity	
Number of shuttles	18.3 (9.3)	18.2 (7.6)
Breathlessness score	6.4 (1.9)	6.7 (2.0)

### Transtricuspid pressure gradient

**Figure 2:**
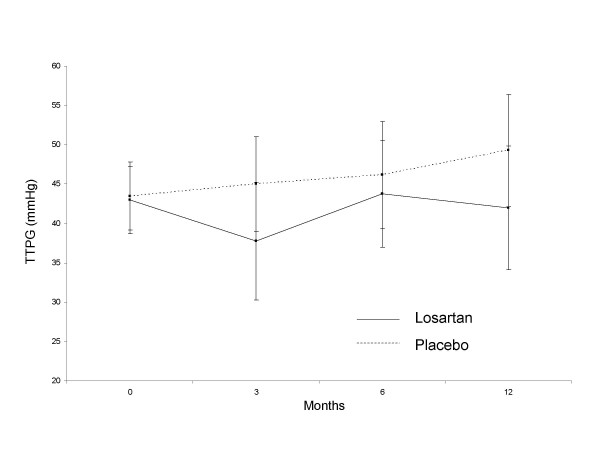
Transtricuspid pressure gradient in placebo and losartan groups. Data points represent means (95% confidence interval).

Measurements of TTPG were similar at -4 weeks and at baseline and all patients had evidence of tricuspid regurgitation and pulmonary hypertension; the mean TTPG at -4 weeks was 44.6 (11.9) mmHg and at baseline was 43.1 (9.2) mmHg. Based on all available data over the course of the study, the TTPG tended to increase in the placebo group and stay constant in the losartan group with mean increases of 5.03 mmHg and 0.84 mmHg, respectively, (Figure 2). The change from baseline in the losartan group minus that in the placebo group (point estimate of treatment difference) at the end of the study was -4.19 mmHg (95% confidence interval (CI): -13.88 to 5.50 mmHg; p = 0.39). The greatest apparent difference between treatment groups was observed at week 12, when the point estimate was -7.49 (95% CI -15.98, 0.99 mmHg) with a p-value of 0.08.

Amongst the losartan-treated group there was considerable variability in the TTPG response to treatment between individual patients, with some patients demonstrating a clinically meaningful reduction in TTPG and in others no response or worsening over the 1-year period. The number of patients completing all assessments in the protocol was less than planned. Taking all available post-randomisation echo's available, the frequency of a reduction in TTPG > 25% at any time point was greater in the losartan group, with 8/16 (50%) than in the placebo group, 4/18 (22%), (p = 0.09).

There were no significant differences between the treatment groups in the changes from baseline in the following secondary efficacy endpoints: right atrial pressure, peak tricuspid regurgitant velocity, right ventricular pressure, left ventricular fractional shortening and right ventricular wall thickness (Table 2).

**Table 2:** Summary of secondary endpoints at baseline and week 48

	**Losartan**			**Placebo**			
	
	**Baseline** **(n = 20)**	**End of study** **(n = 16)**	**Change**	**Baseline** **(n = 20)**	**End of study** **(n = 18)**	**Change**	**p-value**
Right atrial pressure (mmHg)	4.0 (0.8)	4.0 (0.6)	-0.1 (1.0)	4.1 (0.9)	3.8 (0.0)	-0.4 (1.0)	0.56
Peak tricuspid regurgitant velocity (m/s)	3.3 (0.3)	3.2 (0.6)	+0.0 (0.7)	3.3 (0.4)	3.5 (0.5)	+0.2 (0.4)	0.37
RV systolic pressure (mmHg)	46.9 (8.9)	47.3 (14.5)	+0.7 (16.8)	47.5 (10.0)	52.5 (13.4)	+4.7 (10.9)	0.41
LV fractional shortening (%)	33 (8)	36 (7)	+2.5 (8.6)	31 (6)	32 (9)	+1.8 (9.7)	0.84
RV wall thickness (mm)	4.1 (0.8)	4.4 (1.3)	+0.4 (1.1)	4.0 (0.6)	4.2 (0.6)	+0.3 (0.6)	0.69

Between group p-value for the difference in change from baseline.

LV= left ventricular. RV = right ventricular.

**Figure 3:**
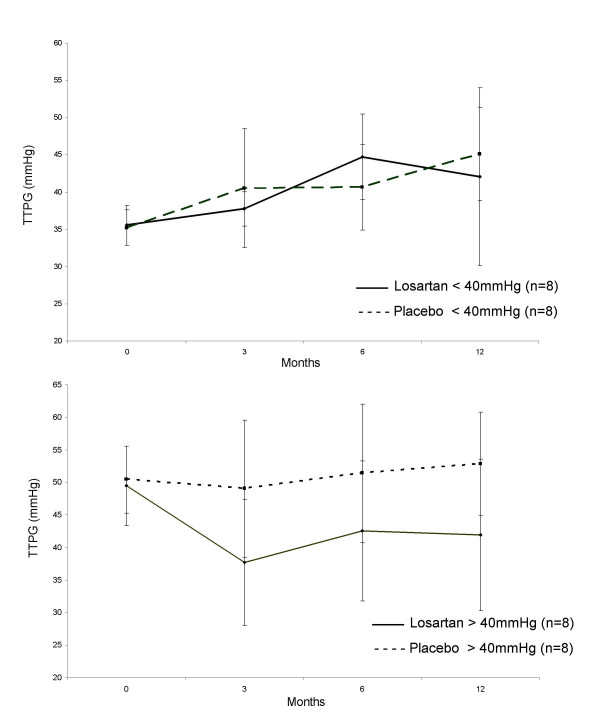
Transtricuspid pressure gradient in placebo and losartan groups, split by baseline TTPG. Data points represent means (95% confidence interval).

Exploratory analysis (Figure 3) suggested a greater treatment effect on TTPG in those patients with baseline >40 mmHg (16% fall on losartan, 4% rise on placebo) than in those patients with baseline TTPG <40 mmHg (30% rise on losartan, 25% rise on placebo).

### Exercise capacity and symptom scores

**Figure 4:**
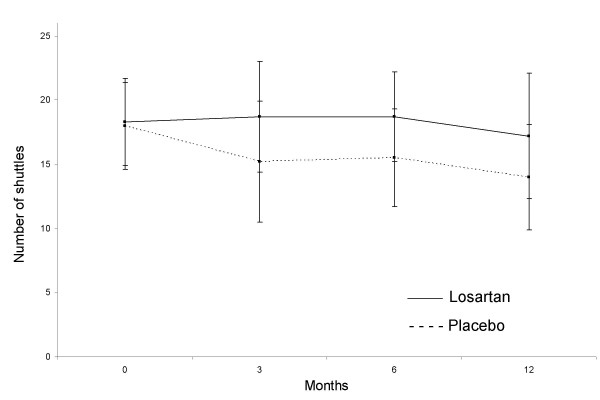
Changes in exercise tolerance measured in placebo and losartan groups by the mean number of shuttles completed in the shuttle walk test. Data points represent means (95% confidence interval).

Symptom-limited exercise capacity tended to stay constant over the course of the study in losartan-treated patients (n = 15); there was a mean decrease from baseline to week 48 of 0.2 shuttles completed (Figure 4). For placebo treated patients (n = 17) there was a mean decrease of 2.6 shuttles completed. Data were available for 32 patients only. No significant treatment differences were found; the between-groups point estimate of treatment difference at the end of the study was 2.39 (95% confidence interval: -1.26 to 6.04) shuttles completed (p = 0.19). Exploratory analysis in those patients with baseline TTPG >40 mmHg failed to suggest a differential effect on exercise capacity.

There were no significant changes from baseline in either treatment group for breathlessness after exercise. For losartan-treated patients, the mean breathlessness score increased from 6.4 (1.9) to 6.8 (1.2) between baseline and week 48; in the placebo group there was a corresponding decrease from 6.7 (2.0) to 6.4 (2.1). The p-value for the point estimate of treatment difference was 0.42. Exploratory analysis in those patients with baseline TTPG >40 mmHg failed to suggest a differential effect on symptom score.

### Quality of life

The St George's Hospital Respiratory Questionnaire produces three component scores (symptom, activity and impact) and an overall score; improvement in quality of life is denoted by a reduction in score. There was a mean decrease of 3.61 in overall score for losartan-treated patients and a mean increase of 1.70 for placebo-treated patients (Table 3). There was no significant treatment difference in the overall score; however, the point estimate of treatment difference approached statistical significance (p = 0.06) in favour of losartan for the activity component of the score.

**Table 3:** Quality of life as assessed on the St George's Hospital Respiratory

	**Losartan**			**Placebo**			
	
	**Baseline** **(n = 19)**	**End of study** **(n = 15)**	**Change**	**Baseline** **(n = 20)**	**End of study** **(n = 18)**	**Change**	p-value*
Overall score	66 (18)	60 (15)	-3.6 (9.2)	65 (14)	66 (19)	+1.7 (9.2)	0.11
Symptom component	72 (17)	67 (22)	-2.7 (15.5)	71 (16)	66 (20)	-5.6 (16.3)	0.61
Activity component	76 (17)	74 (13)	-1.2 (10.8)	77 (17)	83 (19)	+7.5 (14.1)	0.06
Impact component	57 (23)	49 (18)	-5.2 (12.6)	56 (17)	56 (22)	+0.7 (13.1)	0.20

*Between group p-value for the difference in change from baseline.

NB. Improvement in QoL is denoted by a **reduction **in score.

No significant differences were found in any of the dimensions (social, physical, emotional, mental, energy, pain or general health) of the Patient Health Survey (SF-36).

#### Safety

Thirty-five of the forty patients reported at least one adverse event during the study, 16 (80%) in the losartan group and 19 (95%) in the placebo group. One patient (in the losartan group) died from peritonitis due to diverticular disease. Adverse experiences determined by the investigator to be possibly, probably or definitely drug related, were reported for seven patients (35%) in the losartan group and ten patients (50%) in the placebo group. Treatment was discontinued because of a drug-related adverse event by four patients, one in the losartan group (nausea with rash and hypotension) and three in the placebo group (one case each of rash, orthostatic hypotension, dizziness with tremor).

Blood pressure control was not a significant problem. One patient in the losartan group had asymptomatic hypotension (and discontinued treatment); two patients in the placebo group experienced orthostatic symptoms (one discontinued therapy – see above).

There were no adverse changes in arterial blood gases. Mean (sd) PaO_2_for losartan-treated patients was 9.1 (1.2) kPa at baseline and 8.1 (1.3) kPa at study end; the corresponding figures for placebo-treated patients were 8.9 (1.8) and 8.6(1.5) kPa, respectively. For PaCO_2_, mean values were 5.6 (0.8) and 5.8 (0.7) kPa for losartan- and 5.5 (0.7) and 5.5 (1.0) kPa for placebo-treated patients at baseline and study end, respectively.

## Discussion

Despite the encouraging data in animal models of chronic hypoxic pulmonary hypertension [27,28], there have been no previous placebo-controlled long-term studies of angiotensin-receptor antagonists in patients with hypoxic lung disease and pulmonary hypertension. In this pilot study, we were unable to demonstrate any statistically significant beneficial effects of losartan in terms of TTPG, exercise capacity, symptoms or quality of life in the treatment group as a whole. There was an early trend towards improvement in pressure gradient and maintenance of exercise capacity in losartan-treated patients; these changes were not sustained throughout the year-long study. There was a trend to deterioration in the placebo group but differences between treatments did not reach statistical significance. In addition, there was a trend towards an improvement (p = 0.06) in the activity component of the St. George's Hospital Respiratory Quality of Life Questionnaire. Exploratory analysis suggests that patients with more severe pulmonary hypertension (TTPG >40 mmHg) may benefit more than the group as a whole in terms of TTPG reduction, but this did not clearly translate into a clinical benefit. Treatment with losartan in this group of patients was well tolerated with a safety profile comparable to placebo. No safety issue specific to patients with pulmonary hypertension secondary to COPD was identified in this pilot study, in particular no significant effect on arterial blood gases.

The rationale behind this study was based on the findings that angiotensin converting enzyme and angiotensin II are both involved in the pulmonary vascular remodelling associated with hypoxic pulmonary hypertension [13,29] Angiotensin converting enzyme expression is increased in the remodelled arteries of patients with plexogenic pulmonary hypertension [30]. In the hypoxic rat model both captopril, an angiotensin converting enzyme inhibitor, and losartan have been shown to prevent the haemodynamic and structural changes of pulmonary hypertension without inhibiting acute hypoxic vasoconstriction [13,31] In addition, right ventricular remodelling in the chronically hypoxic rat is associated with increased angiotensin converting enzyme expression and activity [32]. Furthermore, angiotensin II stimulates hypertrophy of human pulmonary artery smooth muscle cells in culture [33]. In man, there have been but a few studies of the effect of these agents on pulmonary hypertension; almost all have involved acute administration and variable findings have been reported [14-17,34,35]. The only reported study using losartan in pulmonary hypertension secondary to COPD was also in the acute setting [14]. Oral dosing with losartan (50 mg) produced a significant reduction in mean pulmonary artery pressure and total pulmonary vascular resistance; in addition, plasma aldosterone was significantly lower after treatment with losartan compared to placebo.

It is well recognized that the increased pulmonary vascular resistance in COPD may be due to a combination of reduced cross-sectional area of the pulmonary vascular bed in emphysema, hyperinflation, and hypoxic pulmonary vascular remodelling. We would expect losartan to target only the latter. The relative contribution of these factors almost certainly differs between COPD patients, and there may be a sub-population of patients who stand to benefit more than others.

A number of methodological issues need to be considered before rejecting a beneficial effect. Firstly, fewer patients than planned completed the study, largely due to adverse events unrelated to study medication and more related to the elderly nature of the group. This may have reduced our power. Nonetheless, our pre-study assumptions (baseline TTPG of 46 mmHg, placebo group increase of 3 mmHg per annum and a standard deviation of the change from baseline of 16 mmHg) were not far from those observed (baseline TTPG of 43 mmHg, placebo increase of 5 mmHg per annum and standard deviation of 16.8 mmHg) We conclude therefore that a change of this magnitude is unlikely in the group as a whole. A much larger study would be needed to confidently detect or exclude a smaller effect. There does appear to be a greater proportionate effect on TTPF in patients with higher baseline values raising the possibility of further study in this group. Secondly, the choice of assessments requires consideration. Studies of agents shown to be beneficial such as Iloprost [36] and Bosentan [37] have used right heart catheterisation to assess haemodynamics. The use of echocardiography may be less precise than invasive measurements but affords the opportunity of repeated measurements. We were unable to show an increase in exercise tolerance using the shuttle test which is a maximal test, whereas others have shown benefit using the sub-maximal 6-minute walk. The latter may be more relevant to daily life.

Finally, whilst our study was ongoing, other losartan studies [20,38] using higher doses (50–100 mg) have shown beneficial effects in both diabetic nephropathy and hypertension with left ventricular hypertrophy, raising the possibility that more marked effects may have been seen with a higher dose.

## Conclusion

In conclusion, this pilot study of the effect of losartan 50 mg on pulmonary hypertension secondary to COPD showed no significant sustained differences between control and treatment groups over the course of one year. There was a trend to early benefit in terms of a slowing of the rate of decline of TTPG and exercise capacity which may warrants further study, particularly in patients with more severe disease, and at higher doses.

## List of Abbreviations

AT_1 _Angiotensin II type 1 receptor

COPD Chronic Obstructive pulmonary disease

FEV_1 _Forced expiratory volume in 1 second

FVC Forced Vital Capacity

LV Left ventricle

RAP Right atrial pressure

RA Right atrium

RV Right ventricle

TTPG Transtricuspid pressure gradient

## Conversion Factor

To convert KPa to mmHg, multiply by 7.5

## Competing interests

This study was funded by Merck Sharp & Dohme Ltd. At the time of the work, PR & RB were employees of Merck Sharp & Dohme and may own stock/stock options.

## Authors' contributions

NM, PR, RB were involved in the design of the study. NM, PP, MH, BHS were involved in the acquision of clinical data. NM & PR wrote the manuscript. All authors read and approved the final manuscript.
